# Developing a microfluidic‐based epicPCR reveals diverse potential hosts of the *mcrA* gene in marine cold seep

**DOI:** 10.1002/mlf2.12159

**Published:** 2025-02-20

**Authors:** Wenli Shen, Danrui Wang, Jiangtao Li, Yue Liu, Yinzhao Wang, Xingsheng Yang, Xi Peng, Bingliang Xie, Lei Su, Ziyan Wei, Qing He, Zhiyi Wang, Kai Feng, Wenbin Du, Ye Deng

**Affiliations:** ^1^ CAS Key Laboratory of Environmental Biotechnology, Research Center for Eco‐Environmental Sciences Chinese Academy of Sciences Beijing China; ^2^ Institute for Marine Science and Technology Shandong University Qingdao China; ^3^ Soil Ecology Lab Nanjing Agricultural University Nanjing China; ^4^ State Key Laboratory of Marine Geology Tongji University Shanghai China; ^5^ College of Environmental Science and Engineering Liaoning Technical University Fuxin China; ^6^ Microbiology Division, School of Life Sciences and Biotechnology Shanghai Jiao Tong University Shanghai China; ^7^ State Key Laboratory of Microbial Resources, Institute of Microbiology Chinese Academy of Sciences Beijing China; ^8^ Savaid Medical School University of the Chinese Academy of Sciences Beijing China

**Keywords:** anaerobic oxidation of methane, cold seep, epicPCR, *mcrA* gene

## Abstract

Anaerobic methanotrophic (ANME) microbes play a crucial role in the bioprocess of anaerobic oxidation of methane (AOM). However, due to their unculturable status, their diversity is poorly understood. In this study, we established a microfluidics‐based epicPCR (Emulsion, Paired Isolation, and Concatenation PCR) to fuse the 16S rRNA gene and *mcrA* gene to reveal the diversity of ANME microbes (*mcrA* gene hosts) in three sampling push‐cores from the marine cold seep. A total of 3725 16S amplicon sequence variants (ASVs) of the *mcrA* gene hosts were detected, and classified into 78 genera across 23 phyla. Across all samples, the dominant phyla with high relative abundance (>10%) were the well‐known *Euryarchaeota*, and some bacterial phyla such as *Campylobacterota*, *Proteobacteria*, and *Chloroflexi*; however, the specificity of these associations was not verified. In addition, the compositions of the *mcrA* gene hosts were significantly different in different layers, where the archaeal hosts increased with the depths of sediments, indicating the carriers of AOM were divergent in depth. Furthermore, the consensus phylogenetic trees of the *mcrA* gene and the 16S rRNA gene showed congruence in archaea not in bacteria, suggesting the horizontal transfer of the *mcrA* gene may occur among host members. Finally, some bacterial metagenomes were found to contain the *mcrA* gene as well as other genes that encode enzymes in the AOM pathway, which prospectively propose the existence of ANME bacteria. This study describes improvements for a potential method for studying the diversity of uncultured functional microbes and broadens our understanding of the diversity of ANMEs.

## Impact statement

This study introduces an innovative microfluidic‐based epicPCR technology to investigate the diversity and phylogeny of *mcrA* hosts in deep‐sea sediments. By uncovering a broad range of potential *mcrA* hosts, including archaea and bacteria, this approach helps broaden our understanding of *mcrA* hosts' community biodiversity. The study also reveals distinct distribution patterns of these hosts across sediment layers. It suggests horizontal gene transfer between archaeal and bacterial hosts, highlighting complex genetic interactions that could shape methane cycling in deep‐sea ecosystems. This work provides valuable insights into the microbial dynamics of methane oxidation, with implications for climate and marine ecology.

## INTRODUCTION

Marine cold seep is a unique chemosynthetic habitat where the deeply sourced hydrocarbon‐rich fluids rise to the seafloor and form oases of elevated microbial biomass and various faunal assemblages. In the cold seep sediment, a vast amount of methane is stored in the form of clathrate hydrates. It is vulnerable to mobilization and release into the atmosphere^1^. It is known that methane is a noted greenhouse gas, and its greenhouse effect is 20–30 times more than CO_2_. Hence, preventing methane from escaping into the atmosphere is very important in protecting the environment. The anaerobic oxidation of methane (AOM) in marine ecosystems consumes more than 90% of methane in the seafloor and effectively prevents it from escaping into the atmosphere^2^. The AOM in marine sediments is usually recognized as sulfate‐dependent anaerobic methane oxidation (SAMO), which occurs by dual‐species consortia of an anaerobic methanotrophic (ANME) archaea (ANME‐1, 3, 2a/b/c) and a sulfate‐reducing prokaryote (SRP)^3^. However, the unculturable nature of ANME archaea has posed significant challenges for their study, hindering comprehensive insights into their ecology and function, particularly in complex and extreme environments. Hence, it is crucial to broadly detect and recognize various ANME microbes to enhance our understanding of methane capture and contribute to global climate stability.

Generally, the biochemical pathway of SAMO is operated via a reversal of the methanogenesis pathway, in which, methyl‐coenzyme M (CoM) reductase (MCR) plays a crucial role^4^. ANME microbes use MCR to activate methane to form CH_3_–CoM as the primary intermediate, while methanogen uses MCR to reduce CH_3_–CoM to methane^4^, ^5^. Because of its highly conserved nature, the *mcrA* gene, encoding the α‐subunit of the MCR complex, has been used as a diagnostic marker for detecting both ANME microbes and methanogens^5^. The phylogenies of the *mcrA* gene and 16S rRNA gene have shown to be congruent, leading to the proposal that the *mcrA* gene could serve as an alternative marker to the 16S rRNA gene in the phylogenetic analysis of methanogen populations^5^. While these conclusions primarily derive from research within the ANME archaea, it remains to be fully investigated whether this congruence holds universally in more complex and extreme environments.

Culture‐independent methods of high‐throughput sequencing have become important approaches to evaluating the diversity and abundance of the uncultured ANME microbial community. For instance, sequencing the marker genes of 16S rRNA and *mcrA* aids in the direct and deep understanding of the composition and diversity of the ANME microbial community^2^, ^6^, ^7^, ^8^, ^9^, ^10^. These methods have the advantages of convenience and low cost. However, they cannot overcome the discontinuity between species information and functional information in studying the diversity of the special functional group. This bottleneck is expected to be overcome by an epicPCR (Emulsion, Paired Isolation, and Concatenation PCR) technology, which could fuse a functional marker gene and phylogenetic gene within single cells^11^. In addition, this method has the advantages of high throughput, low cost, and relatively simple analysis, and thus has become a promising method to detect the diversity of specific functional groups in various environments. It has been successfully applied to study the horizontal transfer of antibiotic‐resistance genes, the association of viruses or bacteria with their hosts, and the diversity of SRPs^11^, ^12^, ^13^, ^14^, ^15^, ^16^, ^17^. However, there have been concerns about the uniformity and stability of polyacrylamide (PAM) beads that were generated by a simple vortex method in the epicPCR technology. The PAM beads with large sizes may contain multiple cells and thus lead to the incorrect fusion of the functional and phylogenetic marker genes. Therefore, to accurately apply it to the study of ANME microbes, it is necessary to improve the uniformity and stability of PAM beads.

In the current study, we established a microfluidics‐based single‐cell high throughput sequencing technology (microfluidics‐based epicPCR) and used it to fuse the *mcrA* gene to its hosts' 16S rRNA gene in millions of droplets at one time, to investigate the ANME community in cold seep sediments.

## RESULTS AND DISCUSSION

### Uniform PAM beads generated by the microfluidic chip method

**Figure 1:**
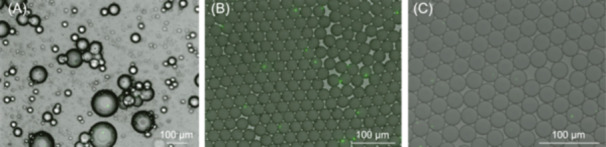
The microscopy images of droplets generated by the vortex and microfluidic chip methods. (A) The microscopy images of the droplets encapsulating fluorescent microspheres generated by the vortex method. (B) The microscopy images of the droplets encapsulating fluorescent microspheres generated by the microfluidic chip method. (C) The microscopy images of the droplets encapsulating sediment samples generated by the microfluidic chip method. The scale bar is 100 μm.

The PAM beads encapsulating the fluorescent microspheres generated by the vortex and microfluidic chip methods are shown in Figure 1. The microscopy images showed that the droplets generated by the vortex method were very heterogeneous and the large‐size droplets usually contained multiple fluorescent microspheres (Figure 1A). Compared to the vortexing method, the droplets generated by the microfluidic chip method had smaller sizes (~30 μm) and were highly uniform (Figure 1B,C), and most of the droplets contained no more than one fluorescent microsphere (Figure 1B). Hence, we encapsulated the cells from the sediment samples using the microfluidic chip method. The droplets containing the encapsulated cells were stained with SYBR Green and observed under an optical microscope. The microscopy images showed that the droplets generated by the microfluidic chip method were uniformly within a diameter of ~30 μm, and the vast majority of them contained no more than one single cell (Figure 1C). The microfluidic chip method has been successfully applied in single‐cell sequencing methods. Zheng et al. published the micro‐seq method, a high‐throughput approach to obtaining individual microbial genomes from complex microbial communities, which encapsulated individual microbes in droplets using the microfluidic chip‐based method and then amplified and sequenced their DNA^18^. In the past decade, the microfluidic chip method has rapidly developed due to its advantages of low cost, high throughput, and ideal separation performance, making it considered the most effective cell isolation method in the development of single‐cell sequencing technologies^19^.

### Diverse potential *mcrA* hosts in marine cold seep

**Figure 2:**
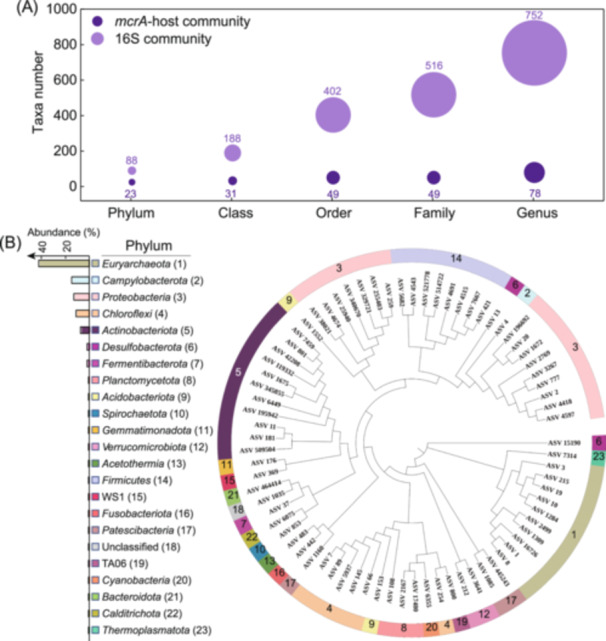
The structure of the *mcrA* gene host community. (A) Statistics of taxa numbers of *mcrA* gene host community and the whole prokaryotic microbial community at different phylogenetic levels. (B) The taxonomic distribution and relative abundances of the *mcrA* gene host community at the phylum level, based on the matched PCR‐16S amplicon sequence variants (ASVs). The numbers following each phylum name indicate their rank in relative abundance and correspond to the labels in the circular phylogenetic tree. The circular phylogenetic tree was constructed using the maximum likelihood method and visualized with iTOL software. The branch lengths in the tree do not represent evolutionary distance.

The composition of the host community was evaluated by the matched PCR‐16S sequences. After removing the 12 amplicon sequence variants (ASVs) of methanogenic archaea, belonging to the genera of *Methanosaeta*, *Methanohalophilus*, and *Methanococcoides* (the only three genera that could be identified as methanogens in this study), a total of 3725 hosts ASVs were classified into 23 phyla, 31 classes, 49 orders, 49 families, and 78 genera. Meanwhile, 120,785 ASVs of the whole prokaryotic community were classified into 88 phyla, 188 classes, 402 orders, 516 families, and 752 genera from the standard PCR‐16S sequences. Out of the entire prokaryotic community, approximately 26.1% of phyla, 16.5% of classes, 12.2% of orders, 9.5% of families, and 10.4% of genera could carry *mcrA* genes (Figure 2A), suggesting that the host species may represent a small fraction of the total ASV diversity within the prokaryotic community. The findings confirmed the dominance of *Euryarchaeota* in methane seep environments, which is consistent with established knowledge in this field (Figure 2B)^20^, ^21^, ^22^. Outside of the well‐known *Euryarchaeota*, some abundant bacterial hosts including *Campylobacterota*, *Proteobacteria*, and *Chloroflexi* were also detected. They showed higher relative abundances of 15.3%, 13.4%, and 12.9%, respectively (Figure 2B). It should be noted that the lack of positive or negative controls to confirm the specificity of our reactions despite all our improvements to the method leaves open the possibility that these novel hosts could be the result of nonspecific associations. Alternatively, this finding may suggest that in the extreme conditions of marine cold seeps, the bacteria might acquire the *mcrA* gene via horizontal gene transfer (HGT), enabling them to participate in methane metabolism. This discovery aligns with emerging evidence that certain bacteria are capable of independently conducting both nitrate reduction and methane oxidation. In 2010, it was discovered that bacteria from the NC10 phylum can independently perform the denitrifying anaerobic methane oxidation (DAMO) process^23^. More recently, in 2023, an anaerobic methane‐oxidizing bacterium was isolated and confirmed to catalyze the entire AOM process through independent denitrification^24^. These findings may expand our understanding of microbial roles in anaerobic methane metabolism beyond the well‐characterized ANME archaea.

### Depth‐driven variations in host community composition

**Figure 3:**
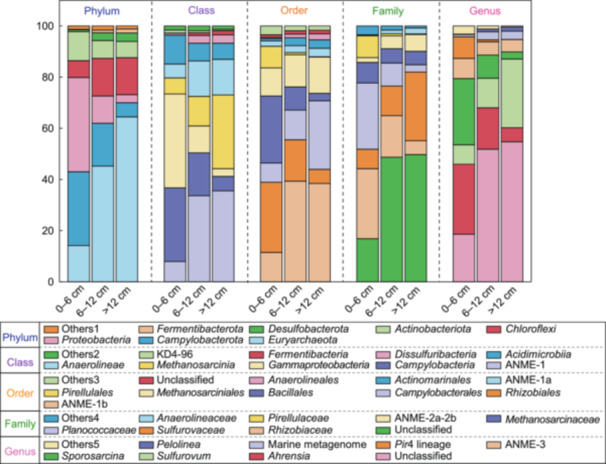
The compositions of *mcrA* gene hosts were identified by epicPCR on fusing *mcrA* and 16S genes in single cells. The different sediment layers showed different trends on the relative abundances at the phylum, class, order, family, and genus levels.

To elucidate the diversity and distribution patterns of host communities within various sediment layers, their richness and relative abundance were compared. The major taxa (>1% relative abundance in at least one layer) of the host community were analyzed at the phylum, class, order, family, and genus level. Except for the unclassified taxa, the hosts primarily belonged to 7 phyla, 9 classes, 9 orders, 7 families, and 7 genera, as shown in Figure 3. The dominant taxa (>10% relative abundance in at least one layer) at the phylum level included *Euryarchaeota*, *Campylobacterota*, *Proteobacteria*, *Actinobacteriota*, and *Choloroflexi*. In general, the relative abundances of hosts belonging to the phyla *Euryarchaeota* increased with depth, whereas the hosts belonging to phyla *Proteobacteria*, *Campylobacterota*, and *Actinobacteriota* showed the opposite trend (Figure 3). These findings revealed significant variances in the host community compositions across the sedimentary layers.

**Figure 4:**
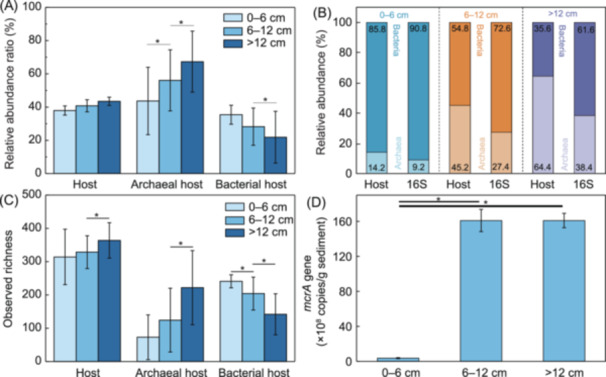
The relative abundance and composition of hosts among different layers. (A) Relative abundance ratios of all hosts (Host), archaeal host community (Archaeal host), and bacterial hosts (Bacterial host) within the total community, archaeal community, and bacterial community, based on the 16S rRNA gene dataset among the different sediment layers. (B) Relative abundances of bacteria and archaea in the host community (Host) and the whole prokaryotic community (16S) among the different sediment layers. (C) The observed richness of all hosts, archaeal hosts, and bacterial hosts among the different sediment layers. (D) The quantities of *mcrA* gene in different depths evaluated by SYBR Green qPCR method. The statistical significance was assessed using the Kruskal‐Wallis test, and significance levels are indicated by asterisks: *p* < 0.05.

The relative abundance of host communities consistently ranged between 37.9% and 43.5% of the total prokaryotic communities across the three sediment layers, showing no substantial variation. However, within the total archaeal population, the archaeal host community displayed a significantly higher relative abundance, spanning from 43.7% to 67.3%, compared to the bacterial host community, which ranged from 21.9% to 35.4% of the overall bacterial population (Figure 4A). Although the total host community's relative abundance remained stable across layers, we observed distinct shifts in the composition of archaeal and bacterial hosts at different depths. This pattern suggests that, despite the stability in overall abundance, specific microbial groups may undergo depth‐related adaptation influenced by unique environmental factors within each layer. Moreover, the relative abundance of archaeal hosts increased with depth, while the relative abundance of bacterial hosts decreased (Figure 4B). According to the observed species richness, archaeal hosts in the 0–6 and 6–12 cm layers were significantly lower than bacterial hosts, while deeper than 12 cm, the archaeal hosts became higher than bacterial hosts (Figure 4C). In addition, the quantity of the *mcrA* gene was significantly higher in the 6 cm deeper layer compared to the 0–6 cm layer (Figure 4D), suggesting that the bacterial hosts nearer to the surface could be playing a much less important role in methane oxidation than archaeal hosts.

Assuming our epicPCR results reveal specific interactions, host communities were distinct at different sediment depths, suggesting that distinct functional microbial groups are responsible for AOM at different depths. The changes in microbial communities in the deep marine sediment were thought to be caused by a combination of organic matter deposition, electron acceptor availability, and sedimentology^25^. A previous report has shown that sediment depth has crucial interactions with the abundance of organic matter and the electron acceptor availability, which would affect the metabolic processes, and thus microbial communities in the deep marine sediment^26^. In addition, the diversity differences of the host community among depth suggested that the distribution of the host community was significantly affected by depth in cold seeps, which is a widely observed pattern for microbial diversity in sediments^10^, ^27^. For example, as depth increases, the abundance of microbes in anoxic marine sediment generally decreases^28^. Moreover, the microbial community in the South China Sea was previously found significantly structured by the sediment age and the availability of electron acceptors. In this study, the increase in archaeal relative abundance and richness with depth was probably due to the fact that archaeal taxa are generally more tolerant of very low energy availability^29^. However, the spatial distribution and environmental constraints of microbial diversity in marine sediments are poorly understood^30^. This study may provide insight into the depth distribution of the host community in cold seep sediments.

### Phylogenetic insights of the *mcrA* gene in cold seep microbial communities

**Figure 5:**
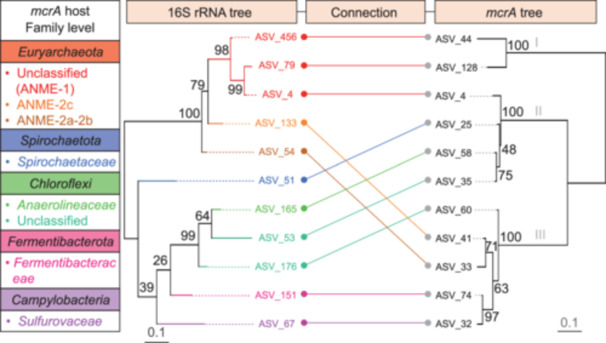
Consensus phylogenetic trees of the *mcrA* gene and its host community (16S rRNA) for all the identified microbial hosts. The tree on the right shows the *mcrA*‐ASVs, and the tree on the left shows the corresponding prokaryotes carrying the *mcrA*‐ASVs. The lines connecting *mcrA*‐ASVs and 16S‐ASVs indicate which 16S‐ASV is the primary carrier of the corresponding *mcrA*‐ASV. The scale bar indicates a 10% sequence divergence both on the left and right.

Taking advantage of the microfluidics‐based epicPCR technology, which created a 730 bp fragment that contained parts of the *mcrA* gene (408 bp), 16S rRNA (298 bp) gene, and two barcodes (12 bp and 12 bp) from a single cell, it was possible to simultaneously analyze both the phylogeny of the *mcrA* gene and the corresponding hosts. Phylogenetic trees were constructed using the most dominant relevant *mcrA*‐ASVs and their corresponding host 16S‐ASVs (Figures 5 and S1). According to the sequence similarity, the *mcrA*‐ASVs were grouped into three clusters, and their hosts were dispersed among five phyla for all identified microbial hosts (Figure 5). All archaeal hosts were known as ANME microbes (ANME‐1 and ANME‐2). This is the first time to our knowledge that bacterial taxa *Spirochaetota*, *Chloroflexi*, *Fermentibacterota*, and *Campylobacteria* have been identified as harboring the *mcrA* gene, although given the lack of demonstrated specificity, additional steps are needed to confirm this. It was found that the hosts of one *mcrA* gene phylogenetic cluster consisted of either only archaeal hosts, such as that of cluster I of the *mcrA* gene, or multiple archaeal hosts with bacterial hosts, such as that of clusters II and III of the *mcrA* gene. Moreover, it was noteworthy that the archaeal host of the *mcrA*‐ASV cluster II belonged only to ANME‐1, while the archaeal hosts‐ASV cluster III belonged only to ANME‐2. Similar results were found in the hosts in different layers. In the 0–6 cm layer, the hosts‐ASV cluster II contained *ANME‐1* and bacteria, and the hosts‐ASV cluster IV contained ANME‐3 and bacteria. In the 6–12 cm layer, the hosts‐ASV cluster III contained ANME‐2a‐2b and bacteria. In the sediment layer deeper than 12 cm, the hosts‐ASV cluster II contained ANME‐1 and bacteria, in addition, the hosts‐ASV cluster V contained unclassified family‐level archaea belonging to *Methanosarciniales* and bacteria (Figure S1).

The phylogenetic trees of 16S rRNA and *mcrA* gene were congruent in archaea but not in bacteria, suggesting that either these are the result of nonspecific associations in the epicPCR pipeline or horizontal transfer of the *mcrA* gene may occur among cold seep community members. We speculated that the frequent contact among microbes in the special habitat of deep marine sediment may lead to the HGT of the *mcrA* gene, although close contact between cells may make it difficult to obtain single cells for our assay. It is known that microorganisms can rapidly adapt to changing environmental conditions by obtaining genes via HGT. The HGT process provides microorganisms a competitive edge and potentially alters the relationship with their host^31^, which may play an important role in the adaptation of microorganisms that inhabit deep‐sea cold seep sediments characterized by extreme darkness, low temperature, and high hydrostatic pressure^32^. Mobile genetic elements (MGEs), which serve to mobilize DNA fragments, have been reported in the anaerobic methanotrophs *Methanosarcinaceae*, indicating the occurrence of HGT^32^. It was found that the HGT of the *mcrA* gene was likely to occur in the bacteria with the ability of hydrocarbon degradation, such as *Chloroflexi*, and *Acidimicrobbiia* of *Actinobacteriota*, or reducing nitrate and thiosulfate, such as *Sulfurovaceae* of *Campylobacterota*. Coincidentally, a few studies have reported that in some cases. Milucka et al.^33^ proposed that some ANME microbes can reduce sulfate without the need for syntrophic partners using a dissimilatory sulfate reduction pathway. Moreover, the AOM‐sulfate reduction (SR) potential was recently found in a single genome of the phylum *Korarchaeota* and the class *Archaeoglobi*, suggesting the possibility that only a single organism may be required for AOM‐SR (sulfate reduction) in some instances^5^, ^10^. Although stringent technical measures were employed to minimize the likelihood of nonspecific fusions, the potential for close cell‐cell contacts leading to such events cannot be entirely ruled out. While our study did not incorporate positive and negative controls to further verify reaction specificity, future work will explore the use of these controls to better exclude this possibility. In addition to cell–cell contact, nonspecific associations could also theoretically form during the nested PCR step if insufficient blocking primers were added, even though we accounted for this in our experimental design by including an adequate amount of blocking primer.

### KEGG pathway maps of hosts

**Figure 6:**
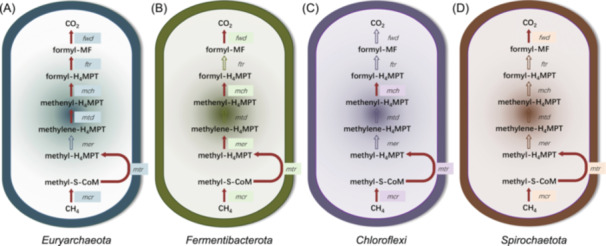
The KEGG pathway maps of anaerobic oxidation of methane in hosts. (A) An archaeal MAG belonging to phylum *Euryarchaeota* (completeness 70.77%). (B) A bacterial MAG belonging to *Fermentibacterota* (completeness 86.74%). (C) A bacterial MAG belonging to phylum *Chloroflexi* (completeness 86.13%). (D) A bacterial MAG belonging to phylum *Spirochaetota* (completeness 78.40%). Solid red arrows indicate the presence of a coding gene for this process, while hollow arrows indicate the absence of the respective functional genes. KEGG, Kyoto Encyclopedia of Genes and Genomes; MAG, Metagenome‐Assembled Genome.

Through metagenomic sequencing, assembly, and binning, a total of 18 archaeal MAGs (Metagenome‐Assembled Genomes) and 80 bacterial MAGs were obtained. According to the annotations from GTDB‐TK (Genome Taxonomy Database Toolkit), these MAGs contained four of five phyla that had been identified as hosts by the microfluidics‐based epicPCR, namely *Euryarchaeota*, *Fermentibacterota*, *Chloroflexi*, and *Spirochaetota*. It is commonly accepted that ANME oxidizes methane to CO_2_ through a reversed hydrogenotrophic methanogenesis pathway^34^; the KEGG maps of AOM (i.e., reverse methanogenesis pathway) are depicted in Figure 6.

The archaeal MAG shown in Figure 6A belongs to the phylum *Euryarchaeota* and has been identified as family ANME‐1, genus QENJ01 by GTDB‐TK. This MAG encompasses all key functional genes of AOM except for *mer* (coding 5,10‐methylenetetrahydromethanopterin reductase). The absence of *mer* has been documented in a previous study because *mer* could be bypassed in ANME‐1^35^. Therefore, this archaeal MAG is considered to be an anaerobic methanotrophic microbe. The bacterial MAGs shown in Figure 6B–D belong to *Fermentibacterota*, *Chloroflexi*, and *Spirochaetota*, and were classified as genus *Aegiribacteria*, order GIF9, and family SLST01 by GTDB‐TK, respectively. These bacterial hosts exhibit partial loss of AOM genes, and none of the bacterial MAGs possesses *mtd* (coding methylenetetrahydromethanopterin dehydrogenase) or *ftr* (coding formylmethanofuran‐tetrahydromethanopterin N‐formyltransferase) gene. Additionally, *Chloroflexi* lacks *mer* and *fwd* (coding formylmethanofuran dehydrogenase) genes, while *Spirochaetota* lacks *mer* and *mch* (coding methenyltetrahydromethanopterin cyclohydrolase) genes. These missing genes are all involved in the lower part of AOM, whereas genes involved in the upper part, namely *mcr* and *mtr* (coding tetrahydromethanopterin S‐methyltransferase), are universally present. Given the potential for chimeras in MAGs^36^, further validation through pure culture experiments is necessary to determine whether the bacterial *mcrA* genes identified in this study truly contribute to methane cycling. While we inferred that these bacterial hosts likely harbor the potential for methane oxidation, they might not be capable of completing the entire process of converting methane to CO_2_ independently. The metagenomes of the bacteria found in this study suggested the presence of possible anaerobic methane‐metabolizing bacteria. However, to confirm whether the putative bacterial *mcrA* gene is genuinely involved in methane cycling, comprehensive metabolic experiments using pure cultures or further metagenomic analysis would be essential. Given the challenges associated with culturing these microbial hosts, this is a complex endeavor that we plan to address in future research.

In this study, we established a microfluid‐based epicPCR technology and applied it to study *mcrA* host diversity and phylogeny. With this new approach, we performed gene analysis at the single‐cell level, providing a new tool for studying the composition of specific functional populations. In addition, we used this microfluid‐based approach to reveal a complex and diverse host community encompassing both archaea and bacteria, with a notable discovery of potential ANME microbes among the *Campylobacterota*, *Proteobacteria*, and *Chloroflexi* phyla, which significantly expanded our knowledge of the diversity of ANME microbes if the specific of these results can be confirmed with additional follow‐up studies. The consensus phylogenetic trees of the *mcrA* gene and the 16S rRNA gene showed congruence in archaea, not in bacteria, which may indicate nonspecific interactions but could also suggest that HGT may play a role in the adaptation of microbes to extreme environments, such as deep‐sea sediments. Furthermore, our study highlights significant spatial variations in host community compositions across sediment layers, underscoring the role of sediment depth in shaping microbial diversity and function in cold seep environments. If our findings can be confirmed, this will deepen our understanding of the ecological role of ANME microorganisms in cold spring environments and provide important clues for further exploration of their metabolic mechanisms.

## MATERIALS AND METHODS

### Collection and preservation of cold seep sediment samples collection and preservation

Three sediment cores, namely HM1, HM3, and HM6, were collected by pushcore from the Haima cold seep in the South China Sea. The samples of HM1 and HM3 were collected from the same batch and were sectioned at 4 cm intervals. In contrast, sample H6 was collected separately and sectioned at 6 cm intervals. The collected samples were stored on dry ice for transport back to the lab and then stored at −80°C until analysis. Samples were prepared in two ways: (1) DNA samples for amplification of the 16S rRNA gene and quantitative PCR of the *mcrA* gene; and (2) cell suspension samples for fusion and amplification of the 16S rRNA gene and *mcrA* gene by epicPCR. DNA samples were extracted from 0.5 g of sediment from the HM1 and HM3 core sections using the DNeasy PowerSoil Pro Kit (Qiagen) according to the manufacturer's instructions. A total of 45 DNA samples were prepared with 10, 20, and 15 replicates for the 0–6 cm (0–4 cm), 6–12 cm (4–8 and 8–12 cm) and deeper than 12 cm (12–16 and 20 cm deeper) layers, respectively. The cell suspension samples were prepared by replicated dilution, vortexing, and sonication of sediment samples from HM1, HM3, and HM6 cores. A total of 43 cell suspension samples were collected with 12, 15, and 16 replicates for the 0–6 cm (HM1/HM3: 0–4 cm and HM6: 0–6 cm), 6–12 cm (HM1/HM3: 4–8/8–12 cm) and deeper than 12 cm (HM1/HM3: 12–16 cm, HM3: 20 cm deeper, and HM6: 12–18/18–24 cm) layer, respectively. The groups were divided based on the samples' community composition and structure. The count of viable cells in the suspension was quantified by adenosine triphosphate (ATP) measurement^37^.

### Cell‐PAM beads produced by the microfluidic chip method

**Figure 7:**
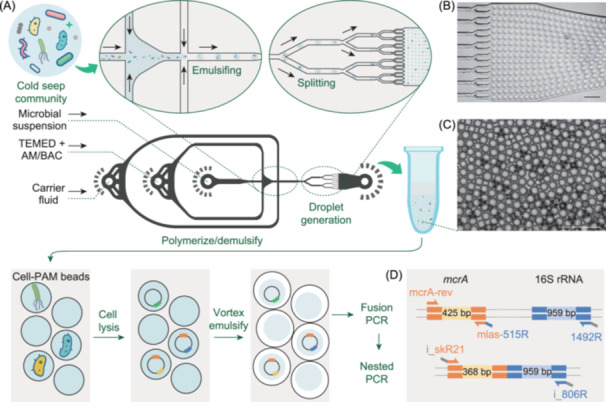
The microfluidics‐based epicPCR workflow for investigation of hosts. (A) Schematic workflow of microfluidics‐based epicPCR with cell‐PAM beads generated by a microfluidic chip. (B) A microscopic image of the splitting region that splits the incoming drops into 16 equal smaller droplets. The scale bar is 100 μm. (C) A microscopic image showing the droplets generated by the chip. The scale bar is 100 μm. (D) The improved epicPCR method lengthened the target functional gene (*mcrA*) fragment. AM, acrylamide; BAC, N,N′‐bis(acryloyl)cystamine; epicPCR, emulsion, paired Isolation, and concatenation PCR; PAM, polyacrylamide; TEMED, N,N,N′,N′‐tetramethylethylenediamine.

To improve the accuracy of fusion PCR, it is crucial to make sure the vast majority of PAM beads contain no more than one single cell. We employed the vortex method used in the original epciPCR technology^11^ and the microfluidic chip method (Figure 7A and Movie S1 in Supporting Information section) to generate PAM beads encapsulating fluorescent microspheres (diameter of 2 μm). In the vortex method, an aqueous suspension, including 30 μl fluorescent microsphere suspension (containing approximately 100,000 fluorescent microsphere), 25 μl 10% (w/v) ammonium persulfate (APS), 200 μl acrylamide solution of 9.4% (w/v) acrylamide (Sigma‐Aldrich), and 0.25% (w/v) N,N′‐Bis(acryloyl)cystamine (BAC; Sigma‐Aldrich), was added to 600 µl emulsion oil in a 2 ml round‐bottom microcentrifuge tube and vortexed for 30 s at 3000 rpm; 25 μl TEMED was added to catalyze the polymerization and vortexed for an additional 30 s at 3000 rpm. After that, the emulsion was polymerized for 90 min, and then the generated droplets were observed under a fluorescence microscope^11^. The microfluidic chip method is as follows. The microchannels were fabricated with polydimethylsiloxane (PDMS) using soft lithography^38^.

The microchannels of the chip consisted of two inlets for aqueous solutions and one for carrier fluid. Syringe pumps were used to drive flows within the chip. The aqueous solutions loaded in two 1‐ml syringes (BD) were (i) a mixture of 30 μl fluorescent microsphere suspension (containing approximately 100,000 fluorescent microsphere), with 70 μl 3.6% (w/v) ammonium persulfate (APS); (ii) 1 ml gel solution consisted of 97.2 μl 24.7% (w/v) acrylamide (Sigma‐Aldrich)/0.66% (w/v) N,N′‐Bis(acryloyl)cystamine (BAC, Sigma‐Aldrich) and 2.8 μl N,N,N′,N′‐Tetramethylethylenediamine (TEMED, Sigma‐Aldrich). The excess carrier fluid (1 ml) loaded in a 5‐ml syringe (BD) was an emulsion oil prepared by suspending span 80 (3% v/v) and EM180 (3% v/v) in hexadecane (Sigma‐Aldrich). Syringes were connected to the chip using 30 gauge Teflon tubing (Zeus). Syringes containing fluorescent microsphere suspension and acrylamide solution were driven by a syringe pump (Pump 11 Pico Plus, Harvard Apparatus) at flow rates of 10 μl/min. The second syringe pump drove the syringe containing the carrier fluid at a flow rate of 40 μl/min. The generated PAM beads were collected with sterile 1.5 ml Eppendorf tubes and observed under a fluorescence microscope (Figure 7B,C). The sediment samples were encapsulated by the microfluidic chip method as described above. Then the generated PAM beads were washed twice with water‐saturated diethyl ether to remove the carrier fluid and rewashed with sterile water at least five times until there was no remaining oil at the top of the tube, before being suspended in 1× TK buffer (20 mM Tris‐HCl and 60 mM KCl) and stored at 4°C until use^11^.

### Design of *mcrA*‐targeted primers in epicPCR

To assess the diversity and phylogeny of the *mcrA* gene and its corresponding prokaryotic hosts, we designed a new *mcrA* gene primer to increase the target length of the *mcrA* gene in the combined *mcrA*‐16S rRNA fragment (Figure 7D). Two pairs of primers were required for targeting the *mcrA* gene in fusion PCR and nested PCR, respectively. The primer pair of mlas (5′‐GGTGGTGTMGGDTTCACMCARTA‐3′) and mcrA_rev (5′‐CGTTCATBGCGTAGTTVGGRTAGT‐3′)^39^ were used in fusion PCR, while the primer mlas and the newly designed primer skR21 (5′‐RCACTGRTCCTGSARGT‐3′) were used in nested PCR. The primer skR21 was designed utilizing the DegePrime program^40^ based on a modified FunGene database (http://mem.rcees.ac.cn/download/modified_FunGene_database_of_mcrA_gene.fasta). Potential hairpin formation and self‐annealing of primer skR21 were excluded by OligoCalc^41^. The coverage of primer skR21 were 62% calculated by an in‐house pipeline (https://ardep.denglab.org.cn/)^42^.

### EpicPCR and 16S rRNA gene amplicon PCR library preparation

The epicPCR procedure was performed by the cell‐PAM bead formation, fusion PCR, AMPure XP bead cleanup, blocking PCR, AMPure XP bead cleanup, nested PCR, and library preparation. Among these procedures, the formation of cell‐PAM beads was conducted using the microfluidic chip method as described above. The fusion PCR, blocking PCR, nested PCR, and AMPure XP bead cleanup were conducted following the published protocols^11^, ^43^ with minor modifications. Fusion PCR amplification was performed using the cell‐PAM beads as the template in ABIL oil (4% ABIL EM90, 0.05% Triton X‐100, mixed by v/v in mineral oil). Reaction agents contained 50 μl 2× HiFi mix (G02C02; Gene‐Protein Link), 2 μl mcrA‐rev primer (50 μM), 2 μl 1492 R primer (50 μM), 1 μl mlas‐515R primer (1 μM), and 45 μl cell‐PAM bead suspension. The thermal cycling protocol consisted of an initial denaturation step at 95°C for 5 min, followed by 35 cycles of 25 s at 95°C, 25 s at 52°C, and 60 s at 68°C. The reaction concluded with a final extension step at 68°C for 10 min. After the cycle was completed, the products were extracted using a mixture of diethyl ether and ethyl acetate, then purified by using Agencourt AMPure XP PCR Purification (Beckman Coulter). The purified products were used to carry out a blocking PCR as follows: mixing 100 μl 2× HiFi mix (G02C02; Gene‐Protein Link), 6.4 μl blocking forward primer (U515F‐block10, 100 μM), 6.4 μl blocking reversed primer (U515R‐block10, 100 μM), and 87.2 μl purified fusion PCR products. The reaction products were purified by Agencourt AMPure XP PCR Purification kit (Beckman Coulter) as above. Then, using the purified blocking PCR products as the template, we carried out the nested PCR, for which the reaction mixture contained 100 μl 2× HiFi mix (G02C02; Gene‐Protein Link), 8 μl skR21 primer (10 μM), 8 μl 806 R primer (10 μM), and 84 μl DNA templates. The amplification conditions were 95°C for 5 min, followed by 35 cycles at 95°C for 25 s, 52°C for 25 s, and 68°C for 45 s, ending with 68°C for 10 min. The product fragment sizes (730 bp) were checked by agarose gel electrophoresis, then collected and purified by Gel Extraction Kit (D2500‐02; OMEGA BioTek) for library construction. According to the MiSeq Reagent Kit Preparation Guide (Illumina 300–300 bp), the libraries were prepared and then sequenced on an Illumina MiSeq platform. The primers used in fusion PCR, blocking PCR, and nested PCR are shown in Tables S1 and S2.

The standard PCR of V4 hypervariable region of 16S rRNA gene was conducted on total microbial DNA of HM1 and HM3 samples using the universal primer pair of 515 F (5′‐GTGYCAGCMGCCGCGGTAA‐3′) and 806 R (5′‐GGACTACNVGGGTWTCTAAT‐3′) combined with self‐designed barcodes as shown in Table S2. The PCR reaction mixture contained 0.5 μl Taq DNA Enzyme (Takara), 5 μl 10× PCR buffer (Takara), 1.5 μl dNTP mixture (10 μM), 1.5 μl forward primer (515 F, 10 μM), 1.5 μl reversed primer (806 R, 10 μM), 1 μl DNA template with a concentration of 20–30 ng/μl, and the volume was adjusted to 50 μl with ddH_2_O. The thermal cycling conditions included an initial denaturation at 94°C for 1 min, followed by 30 cycles of 20 s at 95°C, 25 s at 57°C, and 45 s at 68°C. The reaction was concluded with a final extension at 68°C for 10 min. The purification, library construction, and sequencing procedures of the final DNA products were the same as those for epicPCR products.

### Quantification of *mcrA* gene for prokaryotes at different layers

To assess the gene copy abundances of the *mcrA* gene present in different layers of cold seep marine, real‐time quantitative PCR (qPCR) experiments were performed. The pair of primer mlas (5′‐GGTGGTGTMGGDTTCACMCARTA‐3′) and mcrA‐rev (5′‐CGTTCATBGCRTAGTT NGGRTAGT‐3′) were used to amplify a fragment of *mcrA* gene (~469 bp). Each qPCR reaction was prepared with a total volume of 20 μl, consisting of 10 μl MonAmp™ SYBR Green qPCR Mix, 0.4 μl of each primer (10 μM concentration), 2 μl of template DNA (5–30 ng), and 7.2 μl of nuclease‐free water. Each sample was tested in triplicate. The thermal cycling protocol included an initial denaturation at 95°C for 30 s, followed by 40 cycles at 95°C for 10 s, 56°C for 10 s, and 72°C for 30 s. All reactions were run on a CFX96 Touch Real‐Time PCR Detection System (BioRad).

### EpicPCR and standard PCR 16S rRNA gene amplicon sequences processing

The sequence data were processed using an in‐house amplicon sequencing data analysis pipeline (https://dmap.denglab.org.cn)^44^, ^45^, ^46^, which integrated various bioinformatics tools. The raw sequencing reads of both epicPCR products and standard PCR products for the V4 hypervariable region of the 16S rRNA gene were processed as follows: the forward and reverse sequences were combined using the FLASH program^47^ after barcode identification and primer removal. The sequences were then qualified using Btrim^48^ and trimmed to a length of 150 bp for the *mcrA* gene and 200 bp for the 16S rRNA gene. Then, for the standard PCR amplicons of the V4 hypervariable region of 16S rRNA, an ASV table was generated with the UNOISE pipeline^49^ in USEARCH after trim N processing. The taxonomy of representative sequences from the 16S rRNA gene ASVs was classified by the RDP classifier method^50^ using the SILVA ribosomal RNA gene database 138.1^51^ as the reference database. For the epicPCR sequencing reads, the *mcrA* gene and 16S rRNA gene fragments were linked by matching their sequencing reads with those of the combined sequences. The observed 16S rRNA gene sequences were subjected to a stringent quality control process. Specifically, these sequences were aligned against the NCBI nonredundant (nr) database using the BLAST algorithm. Only sequences with an *E*‐value threshold of 10^−5^ or lower were retained, ensuring the inclusion of highly reliable matches. In addition, the *mcrA* genes were screened by searching the protein sequences translated from their nucleotide sequences with the *mcrA* HMM model downloaded from the FunGene website^52^, ^53^, ^54^. Only the protein sequences validated as *mcrA* genes were retained for subsequent analysis. Thereafter, the remaining 16S rRNA gene sequences and *mcrA* gene sequences were matched to each other by their sequencing reads. The matched sequences, referred to as epic‐16S and epic‐*mcrA*, were clustered into unique sequences using the UNOISE pipeline, and the taxonomy of the representative sequences of the epic‐16S was classified in the same manner as for the standard PCR amplicons of V4 hypervariable region of 16S rRNA (PCR‐16S).

### Ecological and statistical analysis

Representative sequences of the epic‐16S ASVs were blasted with the representative sequences of PCR‐16S ASVs at 97% similarity and 100% identity to choose the matched PCR‐16S ASVs, which were then used to analyze the richness and abundance of the host community. Since the *mcrA* gene can be amplified from both methanogens and ANME microbes, in this study, we filtered out all methanogens by their genus name that were reported as methanogens (*Methanosaeta* ^55^, *Methanohalophilus* ^56^, and *Methanococcoides* ^57^). The alpha diversity analyses of the host community in different layers were conducted by comparing the relative abundance of the host community with the whole prokaryotic community and assessing the relative abundances of archaea and bacteria in the host community and the whole prokaryotic community, as well as observing the richness of the host community. The beta diversity of the host community among different layers was analyzed by nonmetric multidimensional scaling (NMDS) based on Jaccard distance and dissimilarity test on an in‐house pipeline (https://dmap.denglab.org.cn)^44^, ^45^, ^46^.

The sequences of the *mcrA* gene and 16S rRNA gene detected by the epicPCR were used to construct the consensus phylogenetic tree of the *mcrA* gene and its hosts. First, the amplicon sequence variants of the 16S rRNA gene (16S‐ASVs) with a relative abundance of >0.2% were selected. Then, the most dominant ASVs of the *mcrA* gene (*mcrA*‐ASVs) for each 16S‐ASV were determined, subsequently, the most dominant 16S‐ASVs for each *mcrA*‐ASV were determined. Subsequently, only pairs of 16S‐ASVs and *mcrA*‐ASVs that had the highest bidirectional match and at least 150 matching reads were retained. Additionally, to construct the consensus phylogenetic tree of all identified microbial hosts, only associations observed in at least two samples were included. For microbial communities across different layers, we first identified the most dominant *mcrA*‐ASVs associated with each 16S‐ASV, followed by determining the most dominant 16S‐ASVs associated with each *mcrA*‐ASV. Only pairs that showed the strongest bidirectional match were kept. Finally, we selected the 11 highest relative abundance 16S‐ASVs along with their corresponding *mcrA*‐ASVs to construct the consensus phylogenetic tree. The consensus phylogenetic trees of the *mcrA* gene and its hosts were constructed using the Neighbor‐Joining method in MEGA7 (Molecular Evolutionary Genetics Analysis) software, with bootstrap analysis performed using 500 replicates to assess tree reliability.

### Metagenome sequencing

NovaSeq was applied for a total of 20 samples from HM6 at Guangdong Magigene Biotechnology Co., Ltd. The sequencing library was generated using NEB Next® Ultra™ DNA Library Prep Kit for Illumina®, and then assessed using the Qubit® 4.0 Fluorometer and Qsep400 High‐Throughput Nucleic Acid Protein Analysis system. Finally, the Illumina NovaSeq. 6000 platform was utilized for generating 250 bp paired‐end reads. Meanwhile, DNA replicates of each slice were mixed equally, purified using Agencourt Ampure XP beads, and then loaded on the PromethION Flow Cell R9.4 for library preparation. After that, Oxford Nanopore PromethION was utilized for long‐read sequencing.

### Pathway reconstruction

To eliminate duplicate PCR reads, FastUniq (version 1.1) was employed, while raw short reads were processed using Trimmomatic (version 0.36) to trim and filter sequences. The Trimmomatic parameters applied were: ‐threads 16, ‐phred33, LEADING:3, TRAILING:3, SLIDINGWINDOW:4:20, and MINLEN:50. Next, FastQC (version 0.11.8) was used to evaluate the quality of clean reads. After quality control, HM6 0–6 cm, 6–12 cm, and 12–18 cm were assembled using metaSPAdes (version 3.14.1)^58^, while HM6 18–24 cm was assembled using Megahit (version 1.2.9)^59^. Additionally, long reads generated by Nanopore were utilized for hybrid metagenomics assembly using OPERA‐MS (version 0.9.0)^60^.

A series of steps were undertaken to deal with these high‐quality contigs. Initially, binning was carried out using MetaWrap (v1.3.2), with the selection of bins based on criteria of completeness (>70%) and contamination (<10%)^61^. Subsequently, MAGs were dereplicated using dRep (v3.2.2) to remove redundant strain‐level genomes (primary ANI threshold: 0.9; secondary ANI threshold: 0.99)^62^, and the remaining MAGs were annotated using GTDB‐Tk (v1.5.0)^63^. Coding sequences within each MAG were predicted using Prodigal (V2.6.3)^64^, and the enzymatic functions were annotated using CLEAN (v1.0.1)^65^. Enzyme Commission numbers obtained from CLEAN were then converted into KEGG Orthology numbers, and KEGG Mapper^66^, an online analysis tool, was employed to reconstruct the metabolic pathways.

## AUTHOR CONTRIBUTIONS

**Wenli Shen**: Conceptualization (equal); data curation (lead); formal analysis (lead); funding acquisition (equal); investigation (equal); methodology (equal); project administration (equal); writing—original draft (lead); writing—review and editing (lead). **Danrui Wang**: Data curation (equal); formal analysis (equal); investigation (equal); writing—original draft (equal). **Jiangtao Li**: Resources (lead); writing—review and editing (equal). **Yue Liu**: Investigation (equal). **Yinzhao Wang**: Writing—review and editing (equal). **Xingsheng Yang**: Data curation (equal); writing—original draft (supporting). **Xi Peng**: Data curation (supporting). **Bingliang Xie**: Investigation (supporting). **Lei Su**: Resources (supporting). **Ziyan Wei**: Data curation (supporting). **Qing He**: Investigation (supporting). **Zhiyi Wang**: Investigation (supporting). **Kai Feng**: Data curation (supporting). **Wenbin Du**: Data curation (equal); funding acquisition (equal); investigation (equal); writing—original draft (equal); writing—review and editing (equal). **Ye Deng**: Conceptualization (lead); data curation (equal); funding acquisition (equal); project administration (equal); supervision (lead); validation (equal); writing—review and editing (lead).

## ETHICS STATEMENT

No animals or humans were involved in this study.

## CONFLICT OF INTERESTS

The authors declare no conflict of interests.

## Supporting information

Supporting information.

Supporting information.

## Data Availability

The modified FunGene database of the *mcrA* gene for primer skR21 design is available at the https://denglab.org.cn/download/modified_FunGene_database_of_mcrA_gene.fasta. The 16S rRNA gene sequences and *mcrA*‐16S rRNA sequences are available in the Genome Sequence Archive in the National Genomics Data Center (China National Center for Bioinformation) under accession number subCRA022017. The metagenomics data reported in this paper have been deposited in the Genome Sequence Archive in the National Genomics Data Center, China National Center for Bioinformation/Beijing Institute of Genomics, Chinese Academy of Sciences that are publicly accessible at https://ngdc.cncb.ac.cn/gsa (GSA: CRA009925 for NovaSeq and CRA009932 for Nanopore).
