# Supplementary material for: Developing a microfluidic‐based epicPCR reveals diverse potential hosts of the mcrA gene in marine cold seep
Source: mLife. 2025 Feb 20;4(1):70–82. doi: 10.1002/mlf2.12159 (PMC11868836; doi:10.1002/mlf2.12159)
Supplement: Supplementary file 1 — Supporting information. [file MLF2-4-70-s001.docx]

**Supplementary figure and tables**

Fig. S1. Consensus phylogenetic trees of the *mcrA* gene and its prokaryotic host community in the different sediment layers. The trees on the right show the *mcrA*-ASVs, and the trees on the left show the corresponding prokaryotes carrying the *mcrA*-ASVs. The lines connecting *mcrA*-ASVs and 16S-ASVs indicates the 16S-ASV is the primary carrier of the corresponding *mcrA*-ASV. Scale bar indicates 10% sequence divergence both on the left and right.

Table S1. Sequences of primers used in fusion PCR, blocking PCR and nested PCR processes.

Table S2. Barcodes (5’->3’) for epicPCR and 16S rRNA amplicons.


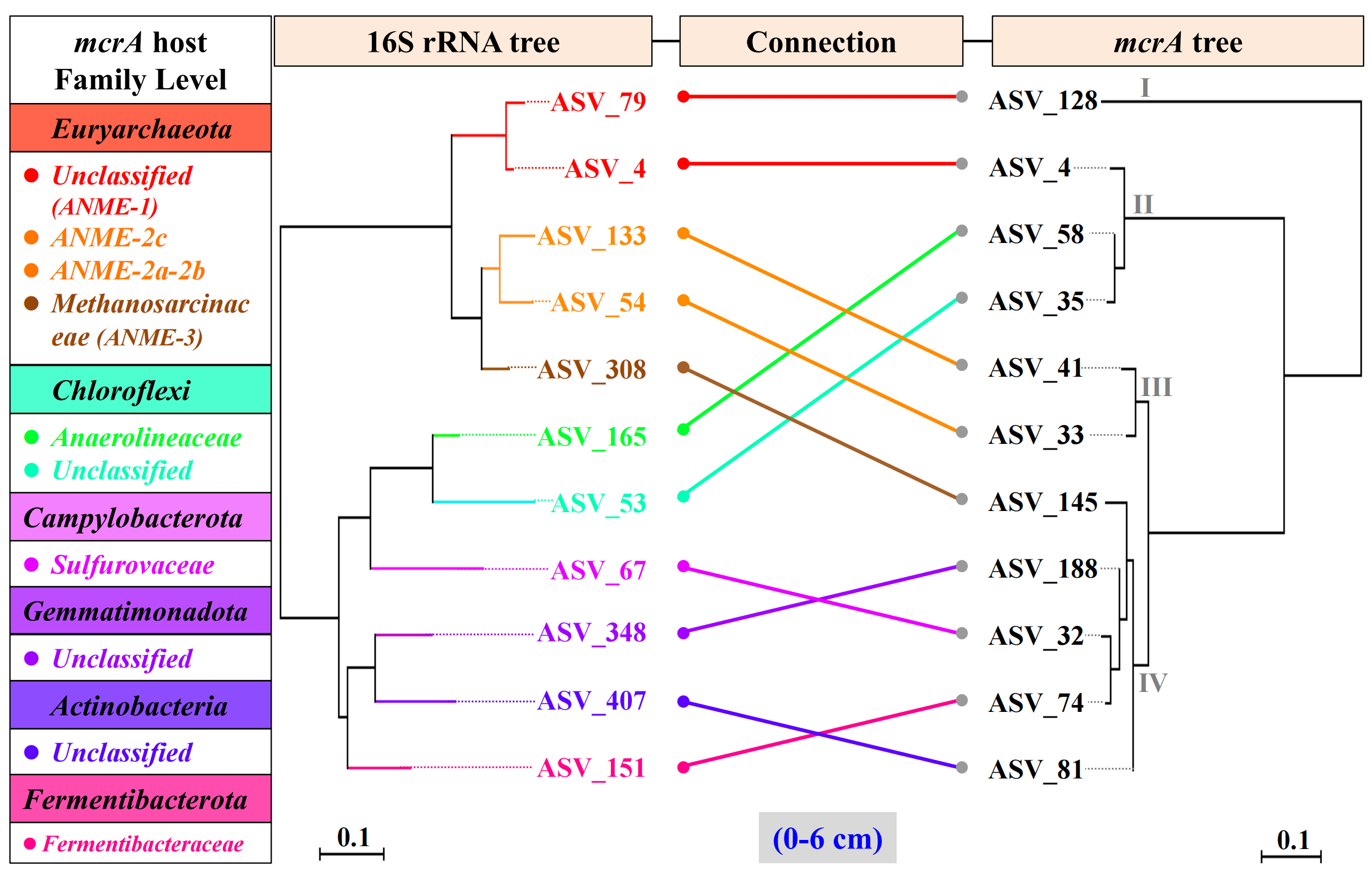


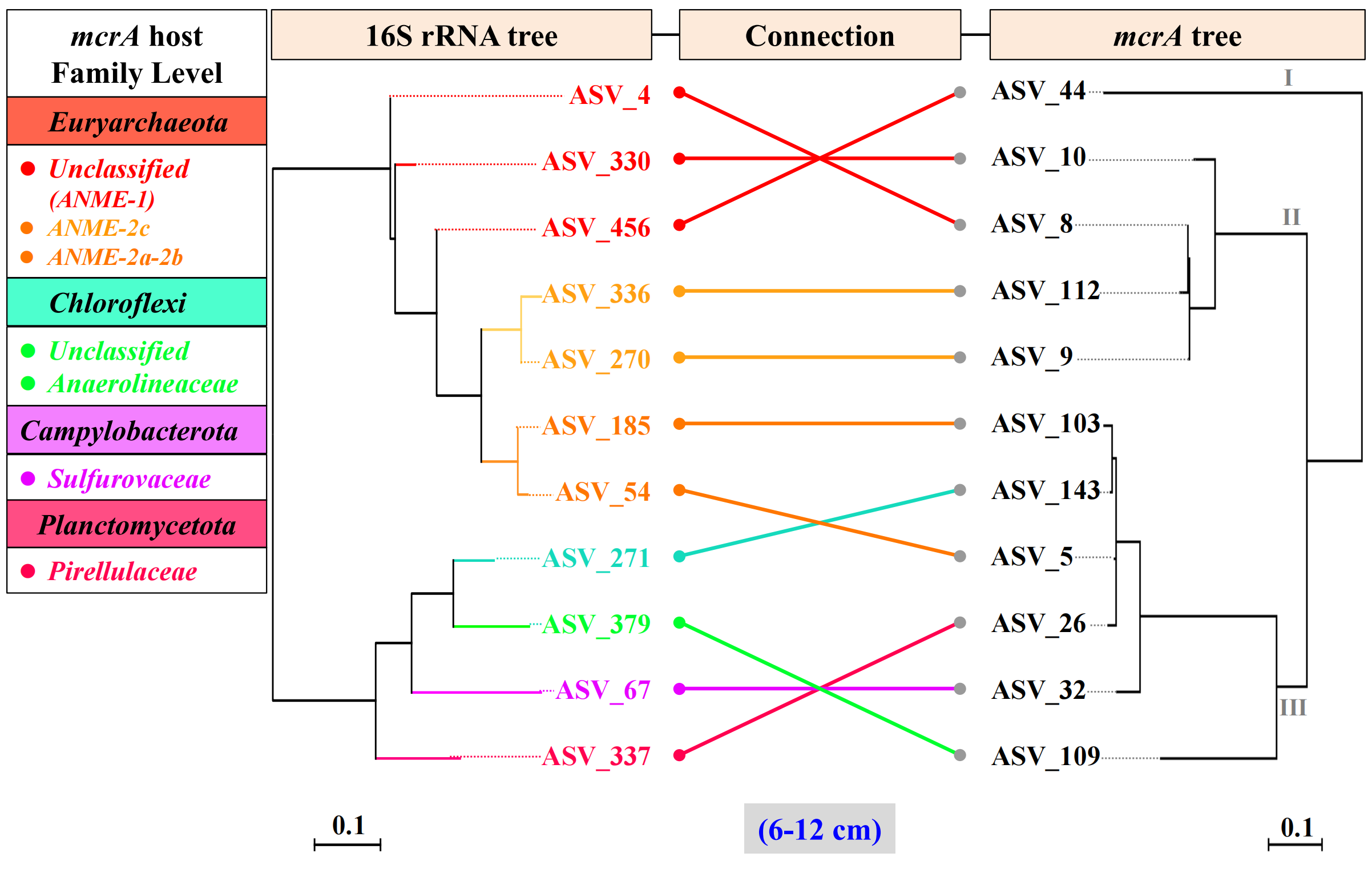

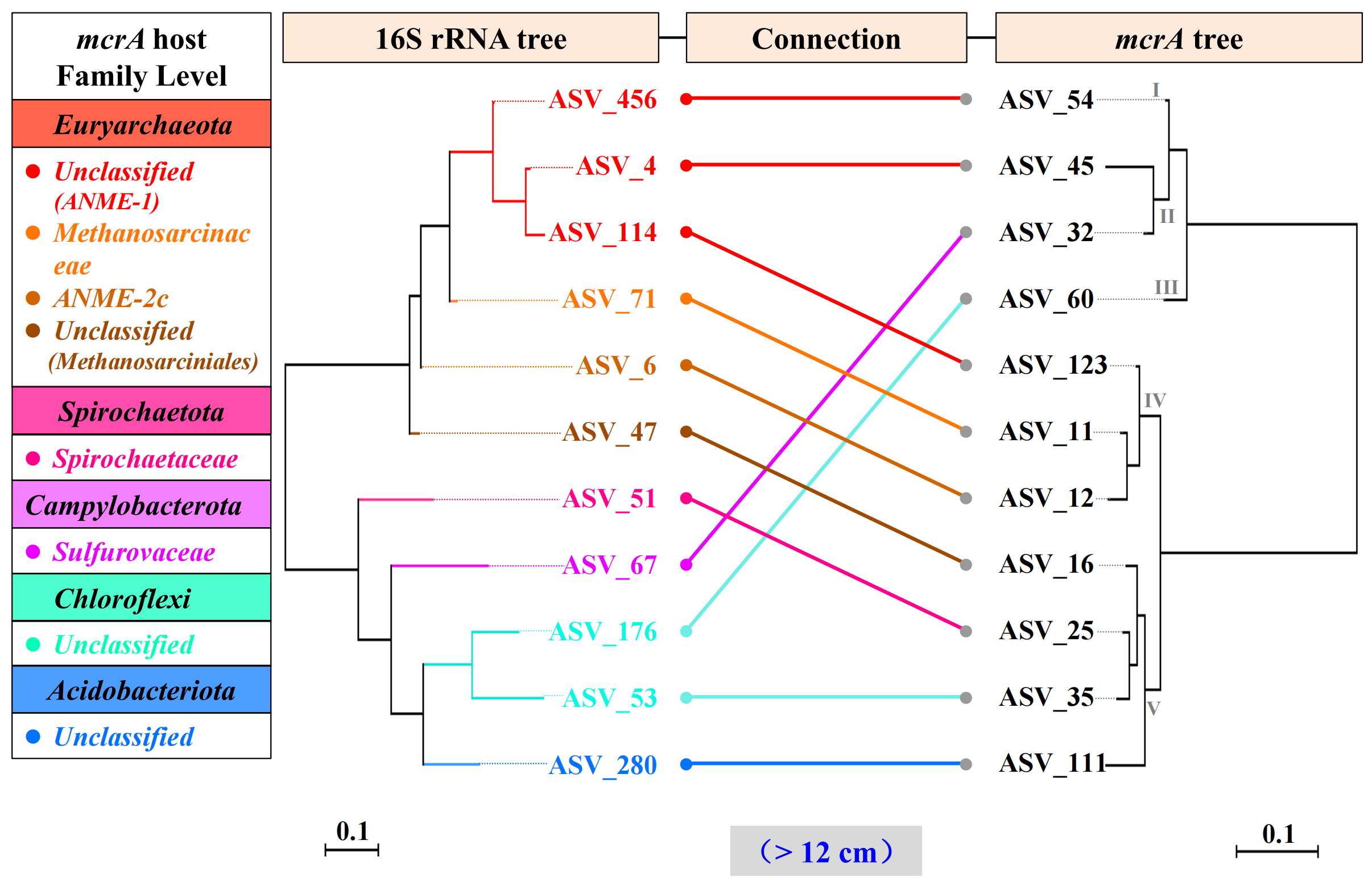


Fig. S1. Consensus phylogenetic trees of the *mcrA* gene and its prokaryotic host community in the different sediment layers. The trees on the right show the *mcrA*-ASVs, and the trees on the left show the corresponding prokaryotes carrying the *mcrA*-ASVs. The lines connecting *mcrA*-ASVs and 16S-ASVs indicates the 16S-ASV is the primary carrier of the corresponding *mcrA*-ASV. Scale bar indicates 10% sequence divergence both on the left and right.

Table S1. Sequences of primers used in fusion PCR, blocking PCR and nested PCR processes

| PCR | Primers | Sequences (5’->3’) |
| --- | --- | --- |
| Fusion PCR | mcrA-rev | CGTTCATBGCRTAGTTNGGRTAGT |
|  | males-515R | TTACCGCGGCKGCTGRCACGGTGGTGTMGGDTTCACMCARTA |
|  | 1492R | GGTTACCTTGTTACGACTT |
| Blocking PCR | U515F-block10 | TTTTTTTTTTGTGYCAGCMGCCGCGGTAA/3SpC3 |
|  | U515R-block10 | TTTTTTTTTTTTACCGCGGCKGCTGRCAC/3SpC3 |
| Nested PCR | skR21 | RCACTGRTCCTGSARGT |
|  | 806R | GGACTACNVGGGTWTCTAAT |

Table S2. Barcodes (5’->3’) for epicPCR and 16S rRNA amplicons

| **Sample** | **epicPCR** | | **16S rRNA** | |
| --- | --- | --- | --- | --- |
|  | forward_barcode  (skR21) | reverse_barcode  (806R) | forward_barcode  (515F) | reverse_bacode  (806R) |
| HM1_0_1 | ATCACATTCTCC | GTCTGTTGAGTG | GAGTCTTGGTAA | AGTCGTGCACAT |
| HM1_0_2 | ATCACATTCTCC | GTCTGTTGAGTG | ATCCTTTGGTTC | AGGGCTATAGTT |
| HM1_0_3 | ATCACATTCTCC | GTCTGTTGAGTG | AATTGTGTCGGA | ACTATGGGCTAA |
| HM1_0_4 | ATCACATTCTCC | GTCTGTTGAGTG | GAATACCAAGTC | GTCACGGACATT |
| HM1_0_5 | ATCACATTCTCC | GTCTGTTGAGTG | GTAGATCGTGTA | GATGTATGTGGT |
| HM1_4_1 | GTTTGCTCGAGA | TCCAGGGCTATA | CAGCTCATCAGC | TGTGCGATAACA |
| HM1_4_2 | GTTTGCTCGAGA | TCCAGGGCTATA | CGAGCAATCCTA | TTGGCTCTATTC |
| HM1_4_3 | GTTTGCTCGAGA | TCCAGGGCTATA | CGAGGGAAAGTC | TGGAGTAGGTGG |
| HM1_4_4 | GTTTGCTCGAGA | TCCAGGGCTATA | AGTTACGAGCTA | GTTGTTCTGGGA |
| HM1_4_5 | GTTTGCTCGAGA | TCCAGGGCTATA | TACGAGCCCTAA | ATGGCTGTCAGT |
| HM1_8_1 | GAAGCTTGAATC | ACGAGACTGATT | CACTACGCTAGA | TGCGCTGAATGT |
| HM1_8_2 | GAAGCTTGAATC | ACGAGACTGATT | GAGCCATCTGTA | GGTGACTAGTTC |
| HM1_8_3 | GAAGCTTGAATC | ACGAGACTGATT | TCGGAATTAGAC | CCACAGATCGAT |
| HM1_8_4 | GAAGCTTGAATC | ACGAGACTGATT | TGTGAATTCGGA | AGTTGAGGCATT |
| HM1_8_5 | GAAGCTTGAATC | ACGAGACTGATT | GGCCAGTTCCTA | GTCGACAGAGGA |
| HM1_12_1 | ACATCAGGTCAC | CAAACGCACTAA | CTATCTCCTGTC | ACTCACAGGAAT |
| HM1_12_2 | ACATCAGGTCAC | CAAACGCACTAA | ATGATGAGCCTC | GAACACTTTGGA |
| HM1_12_3 | ACATCAGGTCAC | CAAACGCACTAA | CATCCCTCTACT | TCGACATCTCTT |
| HM1_12_4 | ACATCAGGTCAC | CAAACGCACTAA | CGGTCAATTGAC | CATCTGGGCAAT |
| HM1_12_5 | ACATCAGGTCAC | CAAACGCACTAA | GTGGAGTCTCAT | CCGAATTGACAA |
| HM3_0_1 | GTATTGGTCAGA | GCTAAGTGATGT | CTGTAGCTTGGC | AGCGGAGGTTAG |
| HM3_0_2 | GTATTGGTCAGA | GCTAAGTGATGT | CCAAGATTCGCC | GATCTAATCGAG |
| HM3_0_3 | GTATTGGTCAGA | GCTAAGTGATGT | TCCCATTCCCAT | CGTGGGCTCATT |
| HM3_0_4 | GTATTGGTCAGA | GCTAAGTGATGT | ATCACATTCTCC | GTCTGTTGAGTG |
| HM3_0_5 | GTATTGGTCAGA | GCTAAGTGATGT | GCTAAAGTCGTA | GACAGAGGTGCA |
| HM3_4_1 | TGTAGTATAGGC | GAGTTAGCATCA | ATGCCTCGTAAG | GTGAGGGCAAGT |
| HM3_4_2 | TGTAGTATAGGC | GAGTTAGCATCA | GTCGGAAATTGT | TGGAGAGGAGAT |
| HM3_4_3 |  |  | ACTAGGATCAGT | GTGACGTTAGTC |
| HM3_4_4 |  |  | GTTTGCTCGAGA | CTGGTGCTGAAT |
| HM3_4_5 | TGTAGTATAGGC | GAGTTAGCATCA | GAAGCTTGAATC | GTTGGTTGGCAT |
| HM3_8_1 | AGCCAGTCATAC | GTGTAGGTGCTT | ACATCAGGTCAC | TCCAGGGCTATA |
| HM3_8_2 |  |  | GTATTGGTCAGA | ACGAGACTGATT |
| HM3_8_3 |  |  | TGTAGTATAGGC | CAAACGCACTAA |
| HM3_8_4 |  |  | AGCCAGTCATAC | GCTAAGTGATGT |
| HM3_8_5 | AGCCAGTCATAC | GTGTAGGTGCTT | ACCGATTAGGTA | GAGTTAGCATCA |
| HM3_12_1 | ACCGATTAGGTA | GCACTGGCATAT | AGAACCGTCATA | GTGTAGGTGCTT |
| HM3_12_2 | ACCGATTAGGTA | GCACTGGCATAT | TCACCCAAGGTA | GCACTGGCATAT |
| HM3_12_3 |  |  | GTAGTGTCAACA | CCGCACTCAAGT |
| HM3_12_4 |  |  | GGAAATCCCATC | GTAGCACTCATG |
| HM3_12_5 | ACCGATTAGGTA | GCACTGGCATAT | TCCTAGGTCCGA | ATCGGGCTTAAC |
| HM3_20_1 | AGAACCGTCATA | GTAGCACTCATG | GCCCAAGTTCAC | ATTCAGATGGCA |
| HM3_20_2 | AGAACCGTCATA | GTAGCACTCATG | ACACAGTCCTGA | GCTGTACGGATT |
| HM3_20_3 | AGAACCGTCATA | GTAGCACTCATG | TAAACCTGGACA | TGGTCAACGATA |
| HM3_20_4 | AGAACCGTCATA | GTAGCACTCATG | ATCCTTTGGTTC | AGGGCTATAGTT |
| HM3_20_5 | AGAACCGTCATA | GTAGCACTCATG | AATTGTGTCGGA | ACTATGGGCTAA |
| HM6_0_2 | TCACCCAAGGTA | ATTCAGATGGCA |  |  |
| HM6_0_3 | TCACCCAAGGTA | ATTCAGATGGCA |  |  |
| HM6_12_2 | GGAAATCCCATC | AGTCGTGCACAT |  |  |
| HM6_18_1 | GCCCAAGTTCAC | AGGGCTATAGTT |  |  |
| HM6_18_4 | GCCCAAGTTCAC | AGGGCTATAGTT |  |  |
